# Personal

**Published:** 1897-05

**Authors:** 


### Personal.

Dr. H. C. Leonhardt has removed from Tonawanda to 228 Goundry street, North Tonawanda, N. Y.

Dr. James F. W. Ross, the distinguished abdominal surgeon of Toronto, has been elected president of the Toronto Athletic Club.

Dr. Martha F. Caul, of Buffalo, has removed from 817 West avenue to 149 Franklin street. Hours: 8–10 a. m., 2–3 and 7–8 p. m. Telephone, Seneca 887.

Dr. C. J. Patterson, formerly of Buffalo, has been appointed junior physician at the Manhattan State Hospital. This appointment is made from the medical civil service list.

Dr. Hobart Amory Hare, professor of therapeutics and materia medica at the Jefferson Medical College, Philadelphia, has been appointed consulting therapeutist to the well-known manufacturing chemists, Messrs. Parke, Davis & Co., Detroit. All questions relating to the practical value of new and old remedies and to the relative merit of various preparations, will be referred to Dr. Hare for an opinion.

Dr. Newton L. Bates, medical director United States Navy, will be stationed at Washington for the coming four years and is announced as the president's physician. Dr. Bates is an alumnus (class of '61) of the medical department of the University of Buffalo, and in the same year was commissioned assistant surgeon in the navy. President McKinley is fortunate in his selection of a medical adviser.

